# O072: A targeted MRSA surveillance in long-term-care facilities - Polish example

**DOI:** 10.1186/2047-2994-2-S1-O72

**Published:** 2013-06-20

**Authors:** J Wojkowska-Mach, M Pobiega, D Romaniszyn, A Chmielarczyk, PB Heczko

**Affiliations:** 1 Chair of Microbiology, Jagiellonian University Medical School, Kraków, Poland

## Introduction

Methicillin-resistant *Staphylococcus aureus* (MRSA) is no longer only a nosocomial pathogen. An understanding of the epidemiology of MRSA in long-term care facilities (LTCF) is essential for preparing effective infection control guidelines not only for LTCFs, but for hospitals too.

## Objectives

The aim of this study was to investigate the prevalence and resistance of MRSA isolates from LTCF-residents and to analyze the potential risk factors for MRSA occurrence (defined as MRSA colonization and/or infection).

## Methods

A 1-day point prevalence study (PPS) and 1-year continuous active surveillance (CS) was carried out on a group of 193 LTCF-residents. The presence of *mec*A and *mup* genes and antimicrobial susceptibility was tested. The genetic diversity of the isolates was compared.

## Results

Overall, 57 residents (26.3%) were colonized with SA, 23 of the isolates (43.9%) were methicillin-resistant. There was 17 cases of infections with the Staphylococcus aureus aetiology, of which 10 (58.8%) were caused by MRSA (8 skin infections and 2 pneumonia). The average age for the population of whom 63.2% were female was 76.2 years. The MRSA prevalence in PPS was 11.9%, in CS - MRSA infection incidence was 5.2%. Factors associated with MRSA presence were: the general status of patients, limited physical activity, wound infections (odds ratio, OR 4.6), ulcers in PPS (OR 2.1), diabetes (OR 1.6), urinary catheterization (OR 1.6) and stool incontinence (OR 1.2). Prevalence of MRSA in the group of residents with limited physical activity was 65.8% (relative risk, RR 12.1). Results of the multivariate analysis showed that age, physical activity impairment and ulcers were significantly associated with the risk of occurrence of MRSA.

## Conclusion

The MRSA occurrence in Polish LTCFs was low. Our data indicate a need to checking MRSA especially in group of residents with limitations of physical activity – i.e. with the highest risk of MRSA. Such targeted surveillance is particularly important in countries with limited resources in infection control. Focus on the high-risk population might be a solution for the cost-effective surveillance. This work was supported by a grant from the Ministry of Science and Higher Education No N N404 047236.

## Disclosure of interest

None declared.

